# Tissue chaperoning—the expanded functions of fetuin-A beyond inhibition of systemic calcification

**DOI:** 10.1007/s00424-022-02688-6

**Published:** 2022-04-11

**Authors:** Stefan Rudloff, Willi Jahnen-Dechent, Uyen Huynh-Do

**Affiliations:** 1 Department of Nephrology and Hypertension, Bern University Hospital, Freiburgstrasse 15, 3010 Bern, Switzerland; 2 Department of Biomedical Research, University of Bern, Freiburgstrasse 15, 3010 Bern, Switzerland; 3 Helmholtz-Institute for Biomedical Engineering, Biointerface Laboratory, RWTH Aachen, University Medical Faculty, Pauwelsstrasse 30, 52074 Aachen, Germany

**Keywords:** Fetuin-A, Mineral chaperone, Biomineralization, Inflammation, Kidney injury

## Abstract

Traditionally, fetuin-A embodies the prototype anti-calcification protein in the blood, preventing cardiovascular calcification. Low serum fetuin-A is generally associated with mineralization dysbalance and enhanced mortality in end stage renal disease. Recent evidence indicates that fetuin-A is a crucial factor moderating tissue inflammation and fibrosis, as well as a systemic indicator of acute inflammatory disease. Here, the expanded function of fetuin-A is discussed in the context of mineralization and inflammation biology. Unbalanced depletion of fetuin-A in this context may be the critical event, triggering a vicious cycle of progressive calcification, inflammation, and tissue injury. Hence, we designate fetuin-A as tissue chaperone and propose the potential use of exogenous fetuin-A as prophylactic agent or emergency treatment in conditions that are associated with acute depletion of endogenous protein.

## Chronic kidney disease and calcification

In the general population, pathological deposition of calcium minerals most often occurs on heart valves, in the myocardium, or in the lumen of microvessels or in the wall of large vessels. The degree of coronary artery calcification is closely linked to cardiovascular incidents, including angina pectoris, myocardial infarction, or stroke [10, 11, 124]. Patients with chronic kidney disease (CKD) suffer from progressive calcification in the cardiovascular system and in soft tissues. It is well recognized that the severity of calcification is predictive of their survival [120, 121]. The finding in a high percentage of CKD stage 2–4 patients that coronary artery calcification preceded the dialysis phase may call for an early start of screening and therapeutic measures [12, 118]. By relating serum characteristics to outcomes in more than 300 hemodialysis patients, Ketteler and colleagues found that decreased serum fetuin-A levels were associated with significantly increased all-cause and cardiovascular mortality [56]. Furthermore, fetuin-A levels were inversely related to CRP levels, which indicated that fetuin-A is a negative acute-phase protein. Smaller studies soon confirmed that the calcification burden was indeed inversely correlated with serum fetuin-A [74, 125]. The lowest serum fetuin-A levels were detected in patients with the calcific uremic arteriolopathy syndrome (calciphylaxis) [98], which manifests as painful ulcerated skin lesions associated with widespread cutaneous arteriolar calcification. Further hallmarks of calciphylaxis include local tissue ischemia and cell death, and high mortality rates with an estimated 1- or 5-year survival of 45% or 35%, respectively. As calciphylaxis also triggers a strong inflammatory reaction, it remains unresolved if fetuin-A insufficiency might be cause or consequence of this severe calcification disease. The connection between calcification, serum fetuin-A, and mortality is less well defined in patients with normal renal function: on the one hand, in nearly 1000 cardiovascular patients without prevalent kidney dysfunction, the Heart and Soul study reported that elevated fetuin-A was associated with the metabolic syndrome and hyperlipidemia, but not with hard outcome factors [85]. On the other hand, we reported in the first prospective longitudinal study that non-dialysis patients with reduced serum fetuin-A had more calcification of the aortic valves [58]. In summary, fetuin-A deficiency may be a crucial factor informing about cardiovascular calcification, inflammation, and mortality in CKD patients on dialysis.

## What makes fetuin-A special?

With about 60 kDa apparent molecular weight on reducing protein gel electrophoresis, fetuin-A is a mid-range sized serum glycoprotein. It was first described in 1944 by Pedersen and because of its high abundance in fetal calf serum (even more than albumin) was named after the Latin word fetus [88]. Fetuin-A has been described in all vertebrates studied [64] and is predominantly synthesized by the liver (> 95%). During fetal development, extrahepatic RNA expression was noted in the choroid plexus and protein expression in all major organs as would be expected from a hepatic plasma protein [30, 73, 116]. In human, fetuin-A was identified by two independent groups [43, 102], representing a major part of the α2-band of serum electrophoresis due to its high concentration in extracellular fluids (0.4–1 g/L). Thus, it was named α2-Heremans-Schmid-glycoprotein (AHSG) by Schultze [103], honoring the original co-discoverers. The gene locus was termed *AHSG* accordingly. Human fetuin-A is subject to extensive posttranslational modifications comprising proteolytic processing from a single chain precursor to the mature circulating two-chain protein [50, 79] complex glycosylation [9, 28, 32], serine and threonine phosphorylation [36, 50, 62, 70, 80, 81], and sulfation [47], which may modulate its biological activity and stability. Together with the related plasma proteins fetuin-B, histidine-rich glycoprotein, and kininogen, fetuin-A belongs to the family of type 3 cystatins. Cystatins are cysteine peptidase inhibitors with key roles in a multitude of physiological and pathological processes. However, no specific target peptidase for fetuin-A has been identified to date, despite documented interactions with several proteases [49]. The promiscuous binding capacity of fetuin-A is not restricted to proteases but ranges from small molecules to entire sporozoites (the infectious stages of plasmodia—the protozoan parasite that causes malaria [52]). Functionally, fetuin-A has been ascribed roles in mineral, lipid, or lectin binding, as well as in antagonizing insulin receptor or transforming growth factor beta signaling [49]. Given the wide array of binding partners and its high expression levels, it is reasonable to conclude that fetuin-A primarily fulfills scavenger/carrier functions. In the following paragraphs, the role of fetuin-A as a multifunctional protein will be discussed with an emphasis on inflammation and mineralization biology.

## Fetuin-A and inhibition of calcification

**Fig. 1:**
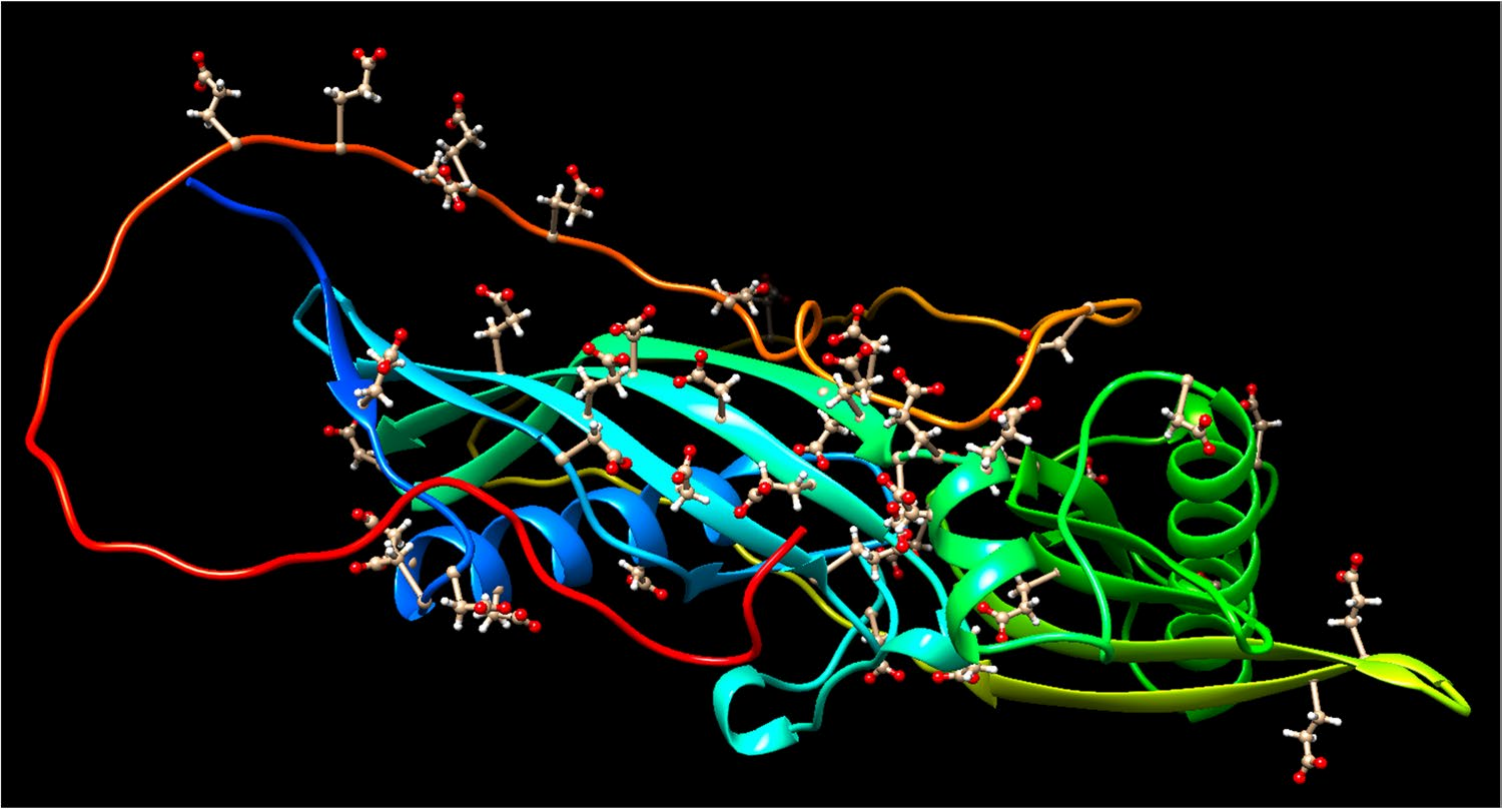
Molecular model of fetuin-A. The model contains the amino-terminal cystatin-like domain 1 CY1 (blue-teal), CY2 (green-yellow), and the carboxyl-terminal region CTR (orange-red) of mouse fetuin-A (UniProtKB—P29699). Acidic residues Asp and Glu are depicted with ball and stick side chains; putative Ser/Thr phosphorylation sites 135, 138, 305, 309, 312, 314, 317, and 320 were replaced by Glu residues in this model. Model generated by AlphaFold2 and depicted by Chimera software [53, 90]

Calcium and phosphate are essential for a multitude of cellular functions and their uptake in the intestine, storage within cells and long-term deposition in bone, and excretion via the kidneys are precisely controlled by the synergistic activity of phosphatonins and calciotropic hormones. With concentrations in the millimolar range, these ions surpass their numerical chemical solubility in water, making the blood a meta-stable calcium phosphate liquid already under physiological conditions. Furthermore, calcium phosphate products such as hydroxyapatite are only marginally soluble in water at physiological pH value. Thus, minimal changes in acid–base homeostasis or ion levels may quickly lead to spontaneous calcification. To counteract the collapse of this finely tuned system, widely disseminated and fast-acting regulatory mechanisms had to evolve that restrict mineralization in space and time to where it is fundamentally required. The confined mineralization of teeth and bones together with the virtual lack of apatite deposition in the systemic circulation indicate that this network of local and systemic calcification inhibitors functions effectively in the general population [48]. A pivotal pathomechanism that tilts the balance of calcium phosphate metabolism could be the loss of one or more of these mineralization inhibitors. CKD patients are especially susceptible to this scenario since the kidneys as one of the central regulators of mineral handling are already functionally impaired. The nature of mineralization inhibitors and the way they prohibit precipitation can be manifold: small molecules like magnesium or pyrophosphate derange the regular crystal structure when incorporated instead of calcium or phosphate, while protein inhibitors often use acidic residues to interfere with the mineralization process. In fetuin-A, the mineral binding site, located in the N-terminal cystatin-like domain CY1, contains multiple negatively charged glutamic acid (Glu) and aspartic acid (Asp) residues arranged in four-pleated beta-sheet [40]. Additionally, phosphorylation of serine (p-Ser) residues located within this structure could further increase the negative charge of the binding interface. Serum fetuin-A phosphorylation analysis reports partial phosphorylation [36, 50], and phospho-fetuin-A was the predominant protein species in both human [108] and rat [68] protein-mineral complexes/calciprotein particles (CPP), suggesting that like in other proteins regulating biomineralization [112], phosphorylation regulates the mineral-binding of fetuin-A. Smith et al. further reported that pre-dialysis CKD patients had accumulated CPP in serum, and that the amount of fetuin-A in these particles was inversely associated with aortic stiffness [108]. The arrangement of p-Ser, Glu, and Asp residues is unique to fetuin-A and lacking in other type 3 cystatin family members [64]. Theoretically, both crystal nucleation and growth can be impaired during the interaction of mineralization-inhibiting proteins and minerals. Using small-angle X-ray scattering (SAXS), an effect of fetuin-A on the nucleation of calcium phosphate crystals was excluded [95]. Different studies employed H_2_O/D_2_O contrast-enhanced small angle neutron scattering (SANS) and found that not ionic calcium but rather small calcium phosphate complexes (Posner clusters) are the preferred fetuin-A ligand. [41, 42]. Furthermore, the lattice constant of these small clusters corresponds nicely to the 6–10 Å distance among the acidic amino acid side chains making up the binding surface in fetuin-A (Fig. 1). Other interfacial analytical methods including differential calorimetry, X-ray analysis, surface probing light scattering, or zeta-potential usually only detected changes in the mineral composition, but not of the interface of the protein. Thus, despite technical advancements, the atomic structure of the interface of fetuin-A and calcium phosphate mineral still has not been resolved, because either the resonance signal of the mineral is much stronger than the protein signal, or the mineral part of the complexes crystalizes much faster than the protein. Welcome help came from the recent publication of the crystal structure of the related protein fetuin-B [24], which allowed us to derive a comprehensive homology model of fetuin-A bound to mineral (Fig. 1). Intriguingly, fetuin-A like other proteins involved in biomineralization has in its carboxy-terminal region CTR a flexible, intrinsically disordered stretch of amino acids that may be crucial for mineral binding [15]. The CTR likely gets displaced when calcium phosphate interacts with the mineral binding domain CY1. An earlier study mapping the mineral-binding motif in fetuin-A likely missed this contribution of CTR to overall mineral binding due to the insensitivity of the available assays [100]. Under mineral supersaturation and low fetuin-A concentration, up to 120 Posner clusters (Ca_9_(PO_4_)_6_) can bind to one protein molecule [42]. We called these smallest basic elements that comprise the majority (95–97%) of the fetuin-A mineral complexes in vitro [42] calciprotein monomers (CPM). In continuous supersaturating conditions, CPM aggregate with each other and bind additional plasma proteins to form larger complexes called calciprotein particles (CPP) [41, 123]. In contrast to CPM, only 3.5% of the fetuin-A, but roughly 50% of the mineral content in solution, is complexed in CPP [42]. By volume, CPP are composed of 75% mineral and 25% fetuin-A. With a diameter of 50–100 nm, primary CPP retain an amorphous morphology and are stable for up to 6 h at 37 °C. Within 24 h, they transform into larger (100–300 nm diameter) secondary CPP with crystallization of the mineral phase into thermodynamically more stable structures, and gradually precipitate thereafter. As would be expected from a colloidal complex, the rate of this 2-stage Ostwald ripening process can be influenced by chemical and physical factors in vitro: Ostwald ripening is accelerated by decreased pH, increased temperature, and the number of freeze–thaw cycles, and increasing the concentration of calcium, phosphate, or lysozyme, while it is slowed by addition of albumin, fetuin-A, or magnesium [72, 86]. Bisphosphonate that has a similar structure to native pyrophosphate, a well-established regulator of calcium phosphate mineralization [84, 106], was recently shown to inhibit not only the aggregation of CPM, but also the phase transition of calcium phosphate, which suggests that both events may be coupled by a yet unknown mechanism [4]. Most importantly however and clinically relevant was the finding that the in vitro kinetics of CPP formation reflect calcification risk in serum of mice and men. A nephelometry-based precipitation assay was developed that estimates the calcification propensity of biological fluids by determining the time of half-maximal transition of primary to secondary CPP [86]. This so-called T50 value precisely reflected the pro-calcifying milieu in fetuin-A deficient mice or calcification-prone hemodialysis patients.

## Metabolism and clearance of calciprotein particles in vivo

**Fig. 2:**
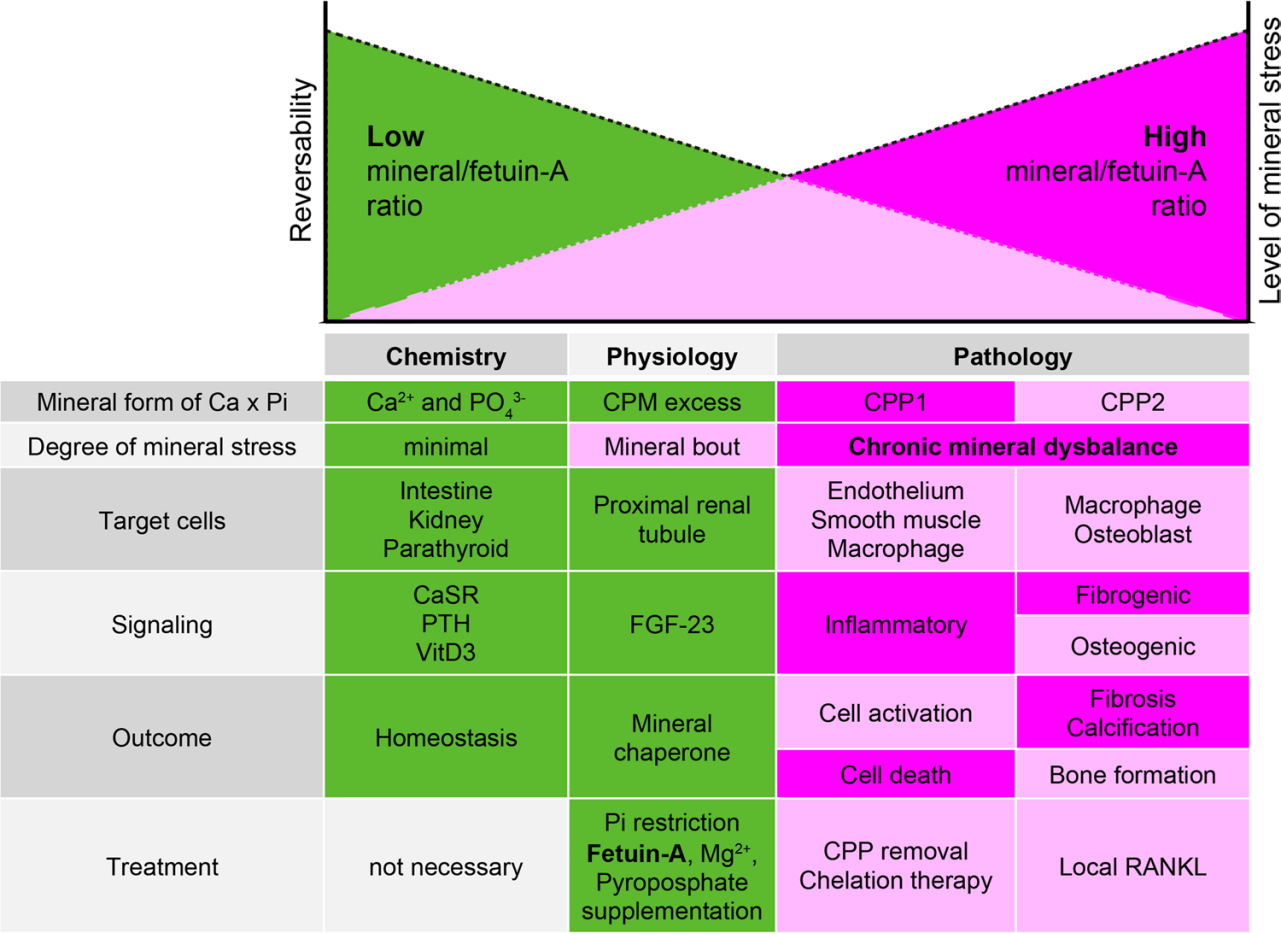
Hierarchical model of mineral-induced stress. The model illustrates the interdependence of the degree of mineral stress and fetuin-A levels, the predominant entities of calcium phosphate mineral particles, their target cells, elicited cellular responses, and possible therapeutic measures. Mineral stress is generally low and reversible under physiological conditions of fetuin-A abundance, when larger, pathological mineral complexes (CPP1 and CPP2) are mostly absent. With decreasing fetuin-A levels, the degree of mineral stress increases due to enhanced formation of CPP. A chronic mineral disbalance eventually leads to irreversible tissue remodeling, inflammation, and calcification

Throughout the years, numerous laboratories reported the formation of CPP-like particles in cultured biological fluids. In 2003, so called fetuin mineral complexes (FMC) were obtained from the serum of rats treated with etidronate [91, 92]. Young and colleagues generated fetuin-A containing granules by incubation of serum-spiked cell culture media with calcium and phosphate [135, 136]. The proposed function of these granules was to control calcium storage, tissue deposition, retrieval, and clearance in conditions of excess calcium phosphate, and thus to inhibit unwanted mineralization in the body. Shortly after the first reports of CPP in vitro, strikingly similar particles were found in the serum of calciphylaxis patients [20, 109], or in the fluid obtained from a patient with calcifying peritonitis [82]. Major drawback of these findings was the prolonged culture time required to observed these particles or the need to “seed” the fluids with external minerals [107]. Thus, speedier isolation protocols were designed to eliminate crystallization artefacts [72, 111]. Indeed, compared to previous studies, the prevailing particles were amorphous CPP1, outnumbering the crystalline CPP2 by tenfold. Given the extensive time the maturation of secondary CPP requires even under supersaturating ex vivo conditions [86], this might not be too surprising at all. Moreover, a flow cytometry-based approach revealed that although CPP were slightly more frequent in dialysis patients compared to healthy controls [110], their number continued to be neglectable in relation to lipoprotein particles or extracellular vesicles that were supernumerary by several orders of magnitude [21, 26]. These findings revealed that CPP are quickly removed from the circulation and that they only form when mineral handling is disturbed. Furthermore, the combination of rapid clearance and the kinetic limitations of CPP ripening make substantial formation of these particles in the blood unlikely. More likely, origins of circulating CPP could be sites where minerals are enriched after dietary intake—the intestine [4, 110]—or where mineralization occurs physiologically—like bone. Here, cryogenic tissue processing methods revealed primary CPP-like particles in the vicinity of caudal fins or rapidly forming long bones in zebrafish or chicken embryos, respectively [55, 67]. In humans, recent findings also suggested a sequential relationship of bone remodeling and serum CPP levels [18]; however, clear data demonstrating that endogenous CPP traffic to and from bone is still missing. The above studies suggest that the level of circulating secondary CPP in vivo is very low, but does not exclude the possibility that fixed within tissues, fetuin-A containing mineral complexes could mature into crystalline secondary CPP-like structures. The strong focus on crystalline CPP for a long time neglected the biological relevance of CPM, even though they are the first line of defense at the mineralization front. With the help of fluorescent fetuin-A, we recently demonstrated that the maturation state and crystallinity of the different calcium mineral particles greatly influenced their processing in vivo. CPM readily passed the renal filtration barrier and fetuin-A—much like albumin—was subsequently resorbed by proximal tubulus cells [57]. In contrast, primary CPP were internalized and rapidly metabolized by hepatic sinusoidal endothelial cells [61]. Like other larger blood particles, secondary crystalline CPP were processed by the mononuclear phagocytic system of the spleen and liver, where they could still be found hours after injection [45, 61]. While the receptors for primary CPP and CPM uptake remain to be identified, secondary CPP are mostly internalized through the class A scavenger receptors (SR-A) with apolipoprotein-A1 acting as a potential ligand [45, 109]. If taken up by macrophages in atherosclerotic plaques, secondary CPP may principally also contribute to plaque calcification [2], which is often observed in CKD patients with disturbed mineral homeostasis. Therefore, the longer fetuin-A bound mineral is retained in vivo and the more time it is given to ripen, the more difficult it becomes to be extracted from the body. Loss of renal function in CKD further reduces CPM clearance and over time leads to an accumulation of mineral-loaded fetuin-A in the body. At first, this may manifest as enhanced CPM levels, which then gradually transform into primary, and in extreme cases of impaired disposal, secondary CPP with all pathological consequences [46, 78, 108]. In vitro, high levels of secondary and especially primary CPP have been shown to activate cells of the innate immune system via toll-like receptor 4 and the NLRP3 (NACHT, LRR, and PYD domains-containing protein 3) inflammasome [57, 61]. If this also pertains to the in vivo situation is a subject of intense research. A hierarchical model summarizing the relationship between mineral-induced stress and the levels of mineral and free fetuin-A is given in Fig. 2.

## Fetuin-A-deficient mice

Total fetuin-A deficiency is likely incompatible with human life. Despite the use of fetuin-A as a forensic marker, complete deficiency has never been reported, except in one recent case report (see below). Given the importance of fetuin-A in mineralization biology, it was therefore surprising to find that fetuin-A-deficient mice do not immediately calcify completely, but instead the degree of ectopic calcification depends on their genetic background. On a C57BL/6 genetic background homozygous deletion of fetuin-A (KO) caused epiphysiolysis, a relatively mild phenotype leading to femur dysplasia and foreshortening of the hindlimbs [19, 104]. This phenotype was reminiscent of Caffey disease, an infantile disorder characterized by excessive new bone formation (hyperostosis), and indeed of a recent case report carrying a nonsense mutation in the *AHSG* gene, which resulted in complete fetuin-A deficiency in the affected child [71]. A completely different phenotype is seen, when fetuin-A was deleted in mice on a DBA/2 background, belonging to one of the most severe extraosseous calcification described [98]. Here, the earliest signs of mineralization were detected in microvascular lumina, which indicated de novo crystallization of calcium-containing microparticles from the liquid phase of blood [44]. The aggregation of these precipitates subsequently caused vascular occlusions, initiating a vicious cycle of ischemia, necrosis, and fibrosis, and further calcifications that readily attained macroscopic dimensions. The calcification phenotype in DBA/2 mice is so severe because of an inherent insufficiency of magnesium and pyrophosphate in this mouse strain. Alone, this double deficiency still suffices to shield the animals from excessive calcification if the animals are kept on a high magnesium diet. When fetuin-A a third extracellular inhibitor of mineralization is deleted, the calcification burden becomes too high [7], and the mice calcify most soft tissues [44, 98]. Half-normal plasma fetuin-A like in hemizygous KO mice suffices to avert this fate even in calcification-prone DBA/2 mice. These findings are particularly relevant to CKD, since the concentrations of fetuin-A and pyrophosphate are frequently reduced in advanced stages of the disease [56, 66]. Plasma magnesium levels on the other hand generally tend to rise with the loss of renal function, and only a fraction of patients presents with inappropriately low levels [134]. Yet again, what matters is not the loss or deficiency of one single mineralization inhibitor, but rather the relationship of all factors combined determines the state of calcification propensity in such patients. Corroborating this view, widespread cardiovascular calcification could also be elicited in partially nephrectomized (to induce CKD) C57BL/6 fetuin-A KO mice on a high phosphate diet [130].

## A protective role of fetuin-A beyond vascular calcification

**Fig. 3:**
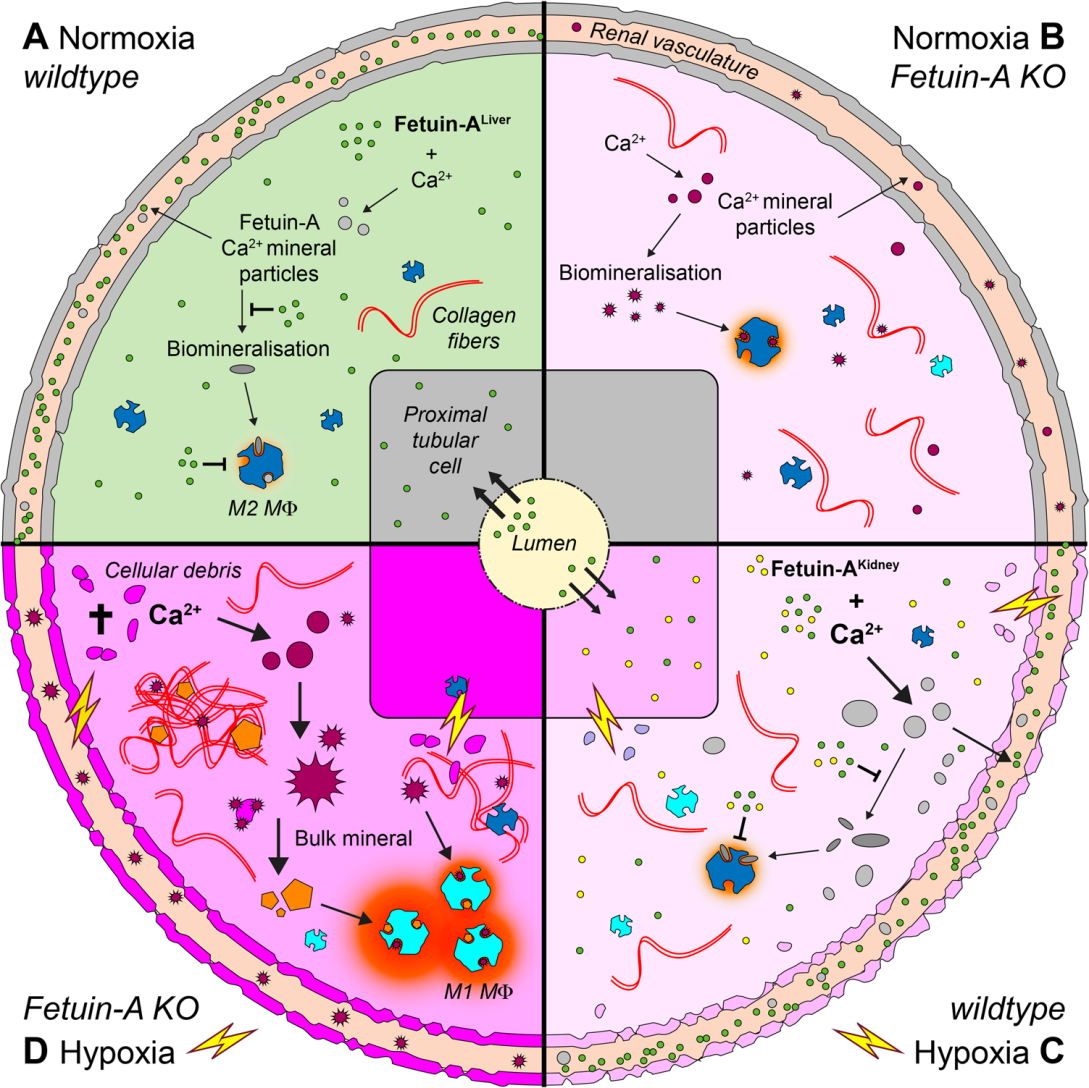
Local tissue protective role of fetuin-A. The model depicts the crucial importance of fetuin-A to safeguard tissue integrity from hypoxia-induced damage in the kidney, through the clearance of calcifying protein-mineral particles, mitigation of inflammation, attenuation of fibrotic tissue remodeling, and polarization of macrophages. (A–D) Clockwise depiction of the 4 different scenarios combining wildtype (WT) or fetuin-A (Ahsg) KO mice with normoxic or hypoxic conditions based on tissue damage intensity: no damage in normoxic WT (A), low damage in normoxic KO (B) and hypoxic WT (C), and strong damage in hypoxic KO (D). In normoxia (A and B) mineral stress is generally low and the absence of liver-derived fetuin-A (green) in B results in slightly elevated fibrotic remodeling. In hypoxia (C and D), mineral stress is generally high due to calcium overload, but extensive tissue damage can be prevented in C by the concerted action of systemic and locally produced fetuin-A (yellow), counteracting calcification and polarization of pro-inflammatory M1 macrophages (M1 MΦ, light blue). Conversely, the absence of fetuin-A in D leads to enhanced calcification, inflammation, and fibrosis

The absence of fetuin-A not only affects the vasculature, but also has profound effects on the integrity of tissues, as we recently showed in a mouse model of Barker hypothesis [96]. According to the Barker hypothesis [8] (also referred to as fetal programming), infants with low birth weight have an increased risk of suffering from cardiovascular disease, high blood pressure, diabetes, and chronic kidney disease in adulthood. During fetal development, protective mechanisms enable adaptation to unfavorable intrauterine conditions (chronic oxygen or nutrient deficiency) and allow for fetal survival. At the same time, however, they lead to permanent structural and functional strains and changes. Using C57BL/6 fetuin-A KO mice exposed to chronic intrauterine hypoxia, we unveiled the importance of fetuin-A during this fetal programming of adult disease phenomenon. As depicted in Fig. 3, fetal hypoxia per se induced fibrotic remodeling, inflammation, and pro-inflammatory macrophage polarization already in the developing kidney of wildtype mice, but this phenotype was exacerbated in fetuin-A-deficient mice [96]. Importantly, in addition to the abovementioned changes, fetuin-A KO fetuses also developed renal microcalcifications, which were not present in wildtype littermates. These lesions likely comprised mineralized cellular debris typically detected at sites of extensive tissue injury or remodeling [35]. These finding suggest that the presence of calcified debris and its ineffective removal initiate a vicious cycle of tissue damage that has profound effects of renal functionality into adulthood. Importantly, the tissue protective role of fetuin-A did not only stem from the liver-derived systemically available fetuin-A but was augmented by extra-hepatic expression of fetuin-A in renal proximal tubular (PT) cells under hypoxic conditions (Fig. 3C). In addition to fetuin-A, approximately one-third of the proteins that make up CPP were also expressed in fetal hypoxic kidneys, including Apo-A1, Apo-A2, and transferrin [111, 131]. Thus, during chronic hypoxia, the fetal kidney invoked salvage mechanisms to minimize the formation and to enhance the clearance of mineralized debris. Cells of the PT are particularly susceptible to straining conditions because they require significant amounts of energy to reabsorb the majority of molecules and ions from the glomerular ultrafiltrate. It is therefore not surprising that these cells are the predominant crystallization sites in the kidney [75], and release proinflammatory cytokines (e.g., MCP1, TNFα, or TGF-β) during cell stress [1, 119]. The hypoxia-induced expression of fetuin-A may in fact indicate a common back-up mechanism in extra-hepatic epithelial tissues that are involved in bulk solute transport to ensure the safe handling of calcium and phosphate in areas of elevated mineral stress [109]. On the other hand, the robust hepatic fetuin-A production may not be further increasable by hypoxia signaling due to an already strong promoter activation in the liver [132]. One reason for the ectopic fetuin-A expression in hypoxic fetal kidneys may be the extra- and intracardiac bypass mechanisms of the fetal circulation redirecting the oxygen-rich blood preferentially to the heart and brain. Interestingly, the expression levels of fetuin-A in all vertebrates correlate with phases of tissue remodeling during embryogenesis, are highest during the initiation of skeletal mineralization, and recede thereafter [97, 116]. A disturbance such as prenatal hypoxia during this period of already high cellular repatterning likely increases the rate of cell death and vascular dysfunction in the kidney [5, 113, 114, 133]. Dying cells and apoptotic bodies are characterized by calcium overload [13, 14, 93] and the simultaneous lack of ATP and pyrophosphate further increase their calcification propensity. In this regard, fetuin-A was reported to decrease the mineralization stress by its ability to inhibit apoptosis and to enhance phagocytosis of apoptotic debris [51, 94]. Thus, fetuin-A deficiency is associated with enhanced deposition and reduced removal of mineral debris, increasing the risk of calcification. A tissue-protective effect of fetuin-A was also observed in various other disease models in adult rodents. For example, we showed that fetuin-A supplementation decreased fibrotic remodeling upon renal ischemia–reperfusion injury [96] and unilateral ureter obstruction (unpublished results). Fetuin-A also reduced paw edema upon induction of tissue inflammation with carrageenan [83]. In the brain, Wang et al. showed that fetuin-A supplementation reduced ischemic damage after permanent middle cerebral artery occlusion in rats [127]. Heinen et al. also reported a reactivation of fetuin-A upon ischemic brain damage in humans, aiding tissue repair [39]. In addition to its function as vascular calcification inhibitor, the above findings and our results suggest a more general role for fetuin-A as an indirect regulator of calcification, fibrosis, inflammation, and macrophage polarization within tissues.

## Fetuin-A and inflammation

Fetuin-A was shown to be an essential cofactor required to inhibit the expression of the proinflammatory cytokine tumor necrosis factor together with spermidine or its synthetic analogues [128, 138]. The strong fetal expression levels of both fetuin-A and spermine have therefore been claimed to underlie the maternal tolerance of the fetus as “nature’s transplant” [129]. The powerful anti-inflammatory role of fetuin-A was confirmed in multiple in vivo rodent models of inflammation, including lipopolysaccharide-induced miscarriage [29], carrageenan injection [83], cerebral ischemic injury [127], cecal ligation and puncture [65], or chronic fetal hypoxia [96]. Generally, fetuin-A was linked to diminished inflammatory responses and greater survival across all cases, and administration of additional fetuin-A typically improved outcomes. Therefore, fetuin-A may be generally considered as an anti-inflammatory agent [23]. The anti-inflammatory properties of fetuin-A were further strengthened by the demonstration of its potent and specific inhibition of hydroxyapatite crystal-mediated activation of neutrophils [117] and its dose-dependent prevention of neutrophil extracellular traps (NETs) formation [89]. Fetuin-A also attenuated calcium phosphate crystal-induced activation of chondrocytes [31, 37, 38], pro-inflammatory cytokine secretion in monocytes and macrophages via NLRP3 inflammasome formation [76, 77, 87], and human vascular smooth muscle cell death [33]. An anti-apoptotic activity of fetuin-A was described in smooth muscle cells [94], and inhibition of cell-specific responses is generally expected to attenuate the deleterious consequences of local inflammation, cell death, and cartilage degeneration. The inhibitory function of fetuin-A on many proinflammatory compounds [6, 126, 128, 129, 137], its established safeguarding function in different animal models of inflammation [29, 65, 83, 96, 127], and the suppression of crystal-induced neutrophil activation [89, 117] collectively and strongly suggest that fetuin-A plays a protective role in mineralopathies in general [105]. Lebreton and colleagues described in the late 1970s that fetuin-A is among the major negative acute phase proteins [63]. The cytokines TNF-alpha, Il-6, and Il-1beta [25] all transiently reduced hepatic fetuin-A mRNA expression, which was attributed to a shift to short isoforms of the transcription factor C/EBP that could not maintain the basal hepatic promotor activity compared to the long C/EBP isoforms predominating in quiescent liver cells [34]. Thus, in certain situations when the demand for fetuin-A is already high (e.g., removing mineral particles), an acute inflammation may be the tipping point that by further lowering fetuin-A production brings the entire system out of balance, and induces gross calcification. Unlike in healthy subjects, the combined effects of excessive calcification and inflammation may consume large amounts of fetuin-A, leading to serum depletion. Especially in patients with CKD stages 4 and 5, the elevated concentrations of uremic toxins and the consequential concomitant chronic inflammation may therefore result in chronic fetuin-A depletion. A rare, but potentially life-threatening clinical presentation of acute fetuin-A depletion in kidney transplant or dialysis patients may be calciphylaxis [16, 101]. Ex vivo analysis showed that sera from these fetuin-A deficient patients could less effectively inhibit crystallization of calcium phosphate minerals compared to healthy controls [98]. Restoring normal fetuin-A levels in these calciphylaxis serum samples by supplementing purified fetuin-A also reversed the functional deficiency. Whether a pre-existing lack of fetuin-A triggers calciphylaxis, or whether plasma fetuin-A becomes depleted by counteracting excessive calcification and, in addition, hepatic expression is repressed by inflammatory cytokines, is presently unclear. Accumulating evidence however suggests that acute injury is another major player contributing to falling serum fetuin-A levels. For example, we recently found in a mouse model of acute kidney injury that systemic fetuin-A levels already rapidly dropped in the sham operation control group, which underwent midline incision of the abdominal cavity, but no renal ischemia reperfusion (unpublished results). This suggests that the acute inflammatory reaction of the wound area is sufficient to cause an intermittent drop in systemic fetuin-A levels. In human patients with acute myocardial infarction, the drop in fetuin-A levels was further shown to correlate with the severity of the myocardial necrosis [99]. Conversely, the level of circulating fetuin-A can also be used to predict incidence and severity of diseases. Dialysis patients within the lowest systemic fetuin-A tertile were much more likely to suffer from stroke than patients in the highest tertile [22]. Furthermore, multiple studies identified low serum fetuin-A associated with of disease severity, hospitalization duration, and mortality of COVID-19 patients [27, 60, 122]. Accordingly, the lowest levels of circulating fetuin-A were found in critically ill sepsis patients [54, 115]. Along these lines, systemic fetuin-A levels reflect acute inflammatory disease states as well as calcification propensity.

## Fetuin-A, a plasma chaperone protein guarding tissues from chronic damage

At first glance, the multifaceted functions of fetuin-A as systemic calcification inhibitor and a crucial factor moderating inflammation and fibrosis in tissue almost seem too much for one single protein. At second glance, the compilation of these roles into one molecular player makes sense, since they all contribute to a common single goal—the protection of the body from harm. The protective role of fetuin-A may be akin skin care products that shield the skin from various harsh environmental conditions. The longer or harsher the conditions, the more skin care must be applied to maintain its protective effect. Especially in sunny, cold, dry, and windy winter conditions, provisional skin care becomes a must if rashes and sores are to be prevented. Fetuin-A fulfills a very similar role in protecting the vascular system and tissues by preventing and mending the cracks and crannies that result from adverse calcification and inflammation. Secondly, fetuin-A is consumed in this process and the magnitude of its depletion seems to correlate with the severity of the damage. However, in contrast to skin care, which can be purchased in any store, while supply lasts, hepatic production of fetuin-A is concomitantly reduced, creating a situation of double shortage. A compelling measure to meet the increased fetuin-A demand is rapid fetuin-A replacement during the crucial phase of tissue injury to compensate for the disseminated calcification consumption and the lack of constant endogenous supply. A suitable source could be the infusion of either human plasma-derived fetuin-A or recombinant fetuin-A protein. Importantly, this intervention does not involve sustained administration of exogenous fetuin-A, but would be an acute treatment, replenishing an outstretched protection system. Main goals are to (1) bridge the gap of insufficient hepatic production, (2) break the microcalcification-borne activation of the inflammatory system, and (3) re-establish the fetuin-A-mediated steady state protection. This does in no way render obsolete tried and true therapeutic approaches in CKD patients. E.g., the phosphate-binding agent sevelamer-HCl increased serum fetuin-A levels in hemodialysis patients, and albeit, elevated fetuin-A levels could first be detected more than 6 weeks after the start of the intervention; they persisted for more than 2 months [17]. In addition to fetuin-A supplementation to counteract acute tissue injuries, these patients should also benefit from anti-inflammatory therapies that may reverse the suppressive effect on fetuin-A expression in the liver. Besides pharmacological interventions or optimization of dialysis, this also applies to relatively inexpensive lifestyle modifications, including personalized physical activity, or various dietary supplementations [3]. Much less clear is the relationship between the degree of calcification, mortality, and fetuin-A levels in patients with normal renal function and in pre-dialysis CKD patients, and high levels of fetuin-A may—according to some association studies—even have detrimental effects on health [69, 85]. Correcting this notion however, a recent Mendelian randomization study co-authored by researchers of one of these earlier studies later did not support a strong, relevant relationship between circulating fetuin-A and diabetes risk in the general population [59]. Presumably, the correlation between high serum fetuin-A and the metabolic syndrome may be simply due to the high caloric intake of these patients, which is known to enhance hepatic protein expression. In summary, we consider fetuin-A a protective protein or “mineral chaperone” protecting injured tissues from calcification and inflammation-related damage. When the calcification propensity exceeds a particular threshold, fetuin-A-dependent compensatory systems eventually will be overwhelmed and the resulting fetuin-A deficiency forms the starting point of a vicious cycle of even more progressive calcification, fetuin-A consumption, tissue destruction, and fibrotic remodeling. Thus, the tissue protective role of fetuin-A applies to all tissues and suggests the use of exogenous fetuin-A as an emergency treatment or prophylactic agent in conditions that are associated with acute depletion of endogenous fetuin-A.
